# Discovery of Potent Dual EGFR/HER2 Inhibitors Based on Thiophene Scaffold Targeting H1299 Lung Cancer Cell Line

**DOI:** 10.3390/ph14010009

**Published:** 2020-12-24

**Authors:** Ranza Elrayess, Yasmine M. Abdel Aziz, Mohamed Saleh Elgawish, Marwa Elewa, Asmaa S. A. Yassen, Sameh S. Elhady, Hosam A. Elshihawy, Mohamed M. Said

**Affiliations:** 1 Pharmaceutical Organic Chemistry Department, Faculty of Pharmacy, Suez Canal University, Ismailia 41522, Egypt; ranza.el-rayes@pharm.suez.edu.eg (R.E.); Marwa_elewa@pharm.suez.edu.eg (M.E.); hosamelshihawy74@hotmail.com (H.A.E.); msaid123eg@yahoo.com (M.M.S.); 2 Medicinal Chemistry Department, Faculty of Pharmacy, Suez Canal University, Ismailia 41522, Egypt; mohamed_elgawish@pharm.suez.edu.eg; 3 Department of Natural Products, Faculty of Pharmacy, King Abdulaziz University, Jeddah 21589, Saudi Arabia; ssahmed@kau.edu.sa

**Keywords:** thieno[2,3-d][1,2,3]triazine, acetamide, H1299, HER2, EGFR

## Abstract

Dual targeting of epidermal growth factor receptor (EGFR) and human EGFR-related receptor 2 (HER2) is a proven approach for the treatment of lung cancer. With the aim of discovering effective dual EGFR/HER2 inhibitors targeting non-small cell lung cancer cell line H1299, three series of thieno[2,3-d][1,2,3]triazine and acetamide derivatives were designed, synthesized, and biologically evaluated. The synthesized compounds displayed IC_50_ values ranging from 12 to 54 nM against H1299, which were superior to that of gefitinib (**2**) at 40 µM. Of the synthesized compounds, 2-(1*H*-pyrazolo[3,4-b]pyridin-3-ylamino)-*N*-(3-cyano4,5,6,7-tetrahydrobenzo[b]thiophen-2-yl)acetamide (**21a**) achieved the highest in vitro cytotoxic activity against H1299, with an IC_50_ value of 12.5 nM in situ, and 0.47 and 0.14 nM against EGFR and HER2, respectively, values comparable to the IC_50_ of the approved drug imatinib (**1**). Our synthesized compounds were promising, demonstrating high selectivity and affinity for EGFR/HER2, especially the hinge region forming a hydrophobic pocket, which was mediated by hydrogen bonding as well as hydrophobic and electrostatic interactions, as indicated by molecular modeling. Moreover, the designed compounds showed good affinity for T790M EGFR, one of the main mutants resulting in acquired drug resistance. Furthermore, both pharmacokinetic and physicochemical properties of the designed compounds were within the appropriate range for human usage as predicted by the in Silico ADME study. The designed compound (**21a**) might serve as an encouraging lead compound for the discovery of promising anti-lung cancer agents targeting EGFR/HER2.

## 1. Introduction

Lung cancer continues to be one of the major causes of cancer-related mortality, and non-small cell lung cancer (NSCLC) cases account for approximately 90% of all lung cancer [1]. Treatment options for patients with advanced lung cancer, including chemotherapy, radiation, or surgery, are still insufficient, and around half of limited-stage lung cancer patients relapse [2,3]. Receptor tyrosine kinases (RTKs) play a significant role in cellular signaling pathways and regulate the majority of cellular processes, such as cell metabolism, differentiation, proliferation, and apoptosis. Among the well-known RTKs, members of the ERbB receptor tyrosine kinase family, including epidermal growth factor receptor (EGFR; HER1/erbB-1), human EGFR-related receptor 2 (HER2; erbB-2/neu), HER3 (erbB-3), and HER4 (erbB-4), represent a promising strategy for targeted therapy in patients with NSCLC due to observed patterns of oncogenic mutations of EGFR and HER2 [4]. Therefore, targeting the ERbB receptor could be a promising strategy in NSCLC eradication. HER2 overexpression currently serves as a biomarker of poor prognosis in many forms of human cancer and is considered to be responsible for around 11–32% of NSCLC tumors, with increased gene copy numbers recognized in 2–23% of cases. As HER2 overexpression is found to potentiate EGFR signaling, dual inhibition of these two pathways is of great clinical interest [5].

**Figure 1:**
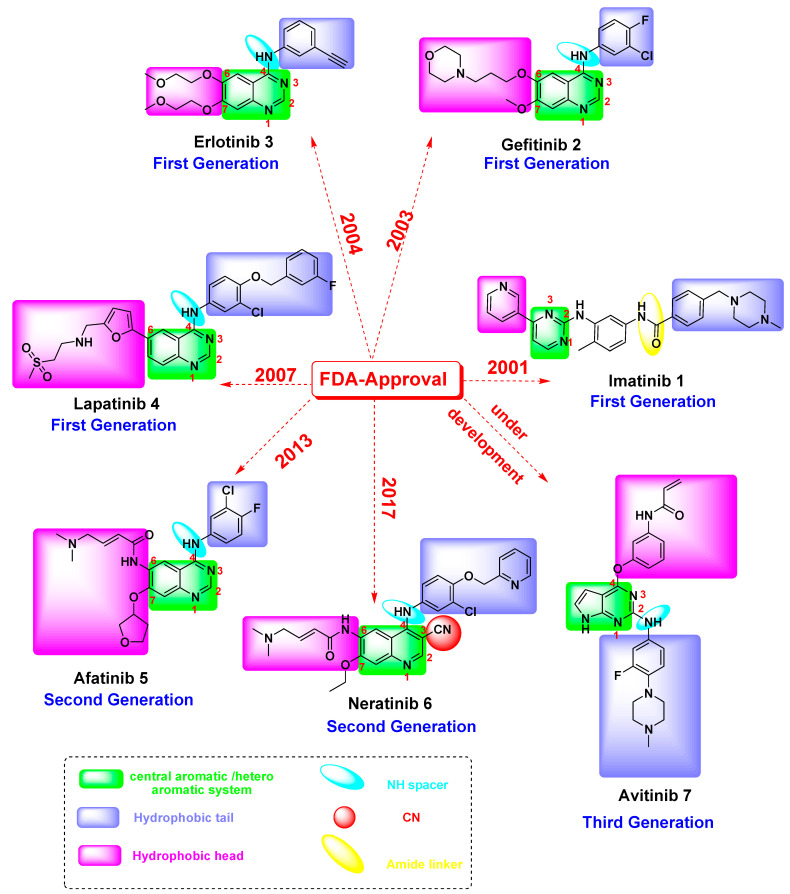
Representation of chemical structures of first-, second-, and third-generation tyrosine kinase inhibitors (TKIs).

First-generation EGFR tyrosine kinase inhibitors (TKIs) such as gefitinib (**2**), imatinib (**1**), lapatinib (**4**), and erlotinib (**3**) have been approved by the US Food and Drug Administration (FDA) for treatment of EGFR-activating mutation-positive NSCLC patients (Figure 1). Acquired drug resistance, however, often occurs after approximately 12 months of treatment with gefitinib (**2**) or imatinib (**1**) [6,7]. Lapatinib (**4**), the first dual oral inhibitor of EGFR and HER2, was approved by the FDA in 2007 for patients with advanced or metastatic breast cancer (Figure 1). These drugs bind competitively and reversibly to the adenosine triphosphate (ATP) binding site of EGFR/HER2, achieving target selectivity by identifying unique characteristics of specific ATP-binding features [5,6,7]. Unfortunately, the ability of these EGFR inhibitors to successfully treat NSCLC is short-lived in subsequent therapies due to acquired T790M missense secondary mutations in EGFR, contributing to drug resistance in around 50% of patients. The mutation of EGFR T790M restores ATP affinity to levels close to those of wild type EGFR, preventing the first generation of TKIs from binding ATP at higher levels. To overcome this resistance, second-generation TKIs, represented by afatinib (**5**) (Vizimpro™, Pfizer Inc., New York, NY, USA) and neratinib (**6**) (Nerlynx™, Puma Biotechnology Inc.), were developed. They have been reported to be superior to first-generation EGFR TKIs in cases of EGFR mutation [6,7].

The majority of the second generation has been developed by structure-guided incorporation of an electrophilic moiety into inhibitors possessing binding affinity to the target of interest. This electrophilic moiety is designed to be attacked by the highly nucleophilic sulfhydryl of cysteine residues, forming a covalent bond [8]. Initially, covalent inhibitors will bind noncovalently and, then, if the reactive moiety trajectory is suitable, covalent bond formation will take place, permanently deactivating TK activity. High selectivity is one big benefit of covalent kinase inhibitors. Additionally, the covalency could allow for extension of the pharmacodynamic period without for the need for elevated drug concentrations [9,10]. Despite the therapeutic success of first- and second-generation EGFR TKIs, EGFR-TK mutation-related resistance remains a significant clinical problem. Great effort has been made to develop alternative strategies, resulting in third-generation TKIs. Third-generation irreversible EGFR inhibitors containing a Michael acceptor functional group have been developed in order to resolve the problem of second-generation irreversible inhibitors with limited wild type (WT) EGFR activity. These inhibitors have acquired increased ATP binding through irreversible alkylation of a cysteine residue. However, these irreversible inhibitors have so far demonstrated only partial clinical efficacy, except in a few cases [11]. Recently, however, interest in irreversible TK inhibitors has had a resurgence from a risk–benefit perspective. The reactivity of electrophilic moieties must be tuned to prevent rapid in vivo quenching and the formation of protein adducts, ultimately leading to hepatotoxicity and mutagenicity [9,10]. High toxicity and weak binding to mutant kinase are the causes of limited clinical efficacy of third-generation TKIs such as avitinib (**7**). (Figure 1) C797S mutations have been reported in cell-free plasma DNA samples from patients who acquired resistance [12]. These data indicate that the development of drugs that do not depend on covalent reaction with Cys797 is important for potency or selectivity.

**Figure 2:**
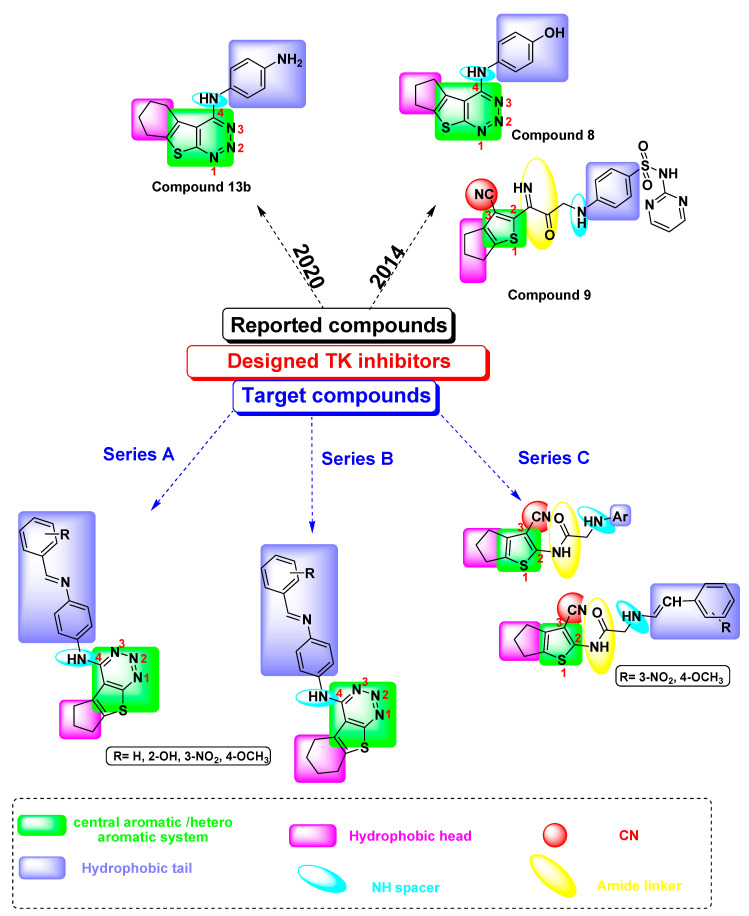
Reported and designed derivatives as dual epidermal growth factor receptor (EGFR)/human EGFR-related receptor 2 (HER2) inhibitors targeting H1299.

Recently, it has been proposed that HER2/EGFR heterodimers or HER2/HER2 homodimers trigger a cascade of signal transduction pathways that are responsible for tumor invasion, progression, and metastasis. Therefore, dual EGFR/HER2 inhibition could be more effective than individual EGFR or HER2 inhibition [13]. In view of this, and in continuing the ongoing strategy aimed at developing new scaffolds for the discovery of potent TKIs, in the current study, a new series of thienotriazine and *N*-(3-cyanothiophen-2-yl)acetamide derivatives was designed and chemically synthesized (Figure 2).

### Rationale and Design

It was found that most of the quinazoline-based anticancer agents that selectively inhibit EGFR/HER2 overexpression develop resistance over a long course of treatment [14,15]. Thus, there is an urgent need to create novel core structures that could replace this quinazoline core, retaining the advantage of the two nitrogen atoms necessary for binding with key amino acids in kinase active sites. In 2014, a study published by our group reported the synthesis of a promising thieno[2,3-d][1,2,3]triazine-based structure as an EGFR inhibitor (**8**) with an IC_50_ of about 38 nM [15]. Moreover, our group designed and synthesized another thiophene-based derivative (**9**) with an IC_50_ of about 30.8 nM against EGFR (Figure 2) [15]. In 2020, our research group succeeded in designing a novel *m*-phenylenediamine thieno[1,2,3]triazine derivative (**13b**) as a promising EGFR inhibitor with an IC_50_ of about 0.33 nM. Compound (**13b**) was targeted to inhibit the growth of the challenging H1299 lung cancer cell line with an IC_50_ of about 25 nM [16]. This success prompted us to develop a novel series of EGFR/HER2 inhibitors targeting H1299.

Moreover, it was observed that the structure of the majority of clinically approved TKIs is characterized by a hydrophobic head and hydrophobic tail linked to a central heteroaromatic system and NH spacer, as shown in Figure 1. A literature survey based on the leading dual EGFR/HER2 targeting compounds lapatinib (**4**) and neratinib (**6**) shows that the upper right warhead, or the hydrophobic tail, is essential for potency of kinase inhibition, while the lower left warhead, the hydrophobic head, is crucial for the dual EGFR/HER2 inhibitory activity. Therefore, to design dual TKIs, we kept the 4-aminoaniline with modified substitution at the meta position as the upper right warhead. On the other hand, the lower right warhead serves as an electrophilic moiety for covalent bond formation in the majority of second- and third-generation agents, and owing to the limited clinical benefit and toxicity, the left warhead was modified in the current study to cyclopentyl and/or cyclohexyl (series A and B) in an attempt to abolish the cytotoxicity. In addition, the hinge region of TKIs, which is occupied by the adenine ring of ATP, was modified to be occupied by thienotriazine in series A and B, and tetrahydrobenzothiophene in series C. Modification of the core scaffold was intended to diminish the cellular resistance common with quinazoline and improve the potency based on our previous publication. Another new modification in this study is a change in the position of the upper warhead from position 4 of quinoline and quinazoline in the majority of approved TKIs to position 2 of tetrahydrobenzothiophene, keeping the hydrophobic tail to mimic the dual EGFR/HER2 inhibitors. The cyano group, which is characteristic of neratinib (**6**), was kept at position 3 to mimic N3 of quinazoline-based TKIs. In this way, various regions of ATP-binding sites could be targeted by such substitution patterns on the mutant EGFR protein kinase domain to design molecules that are differentially selective. The design strategies are displayed in Figure 1 and Figure 2.

## 2. Results and Discussion

### 2.1. Chemistry

**Scheme 1:**
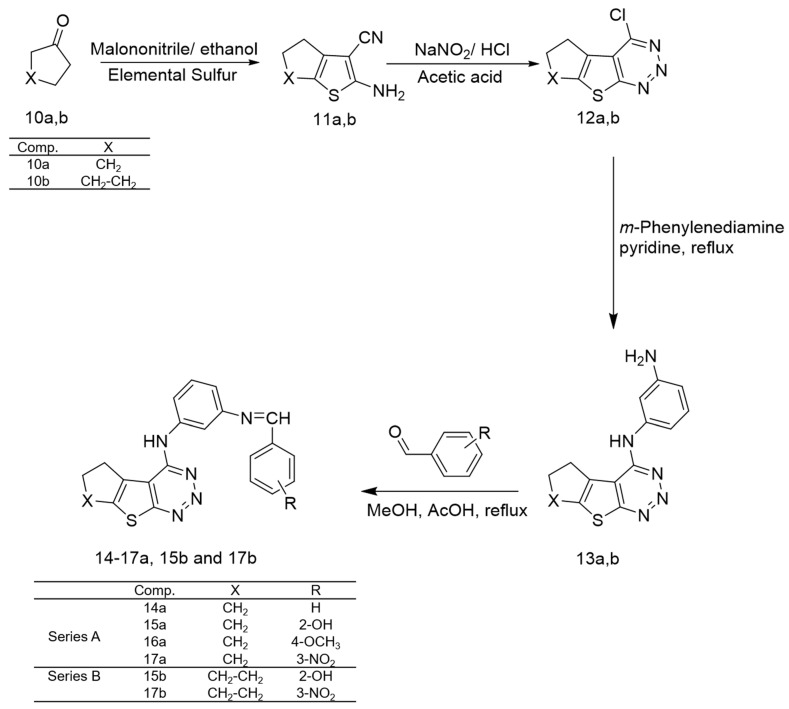
Synthesis of dihydrocyclopentathienotriazine and tetrahydrobenzothienotriazine derivatives (series A, B).

**Scheme 2:**
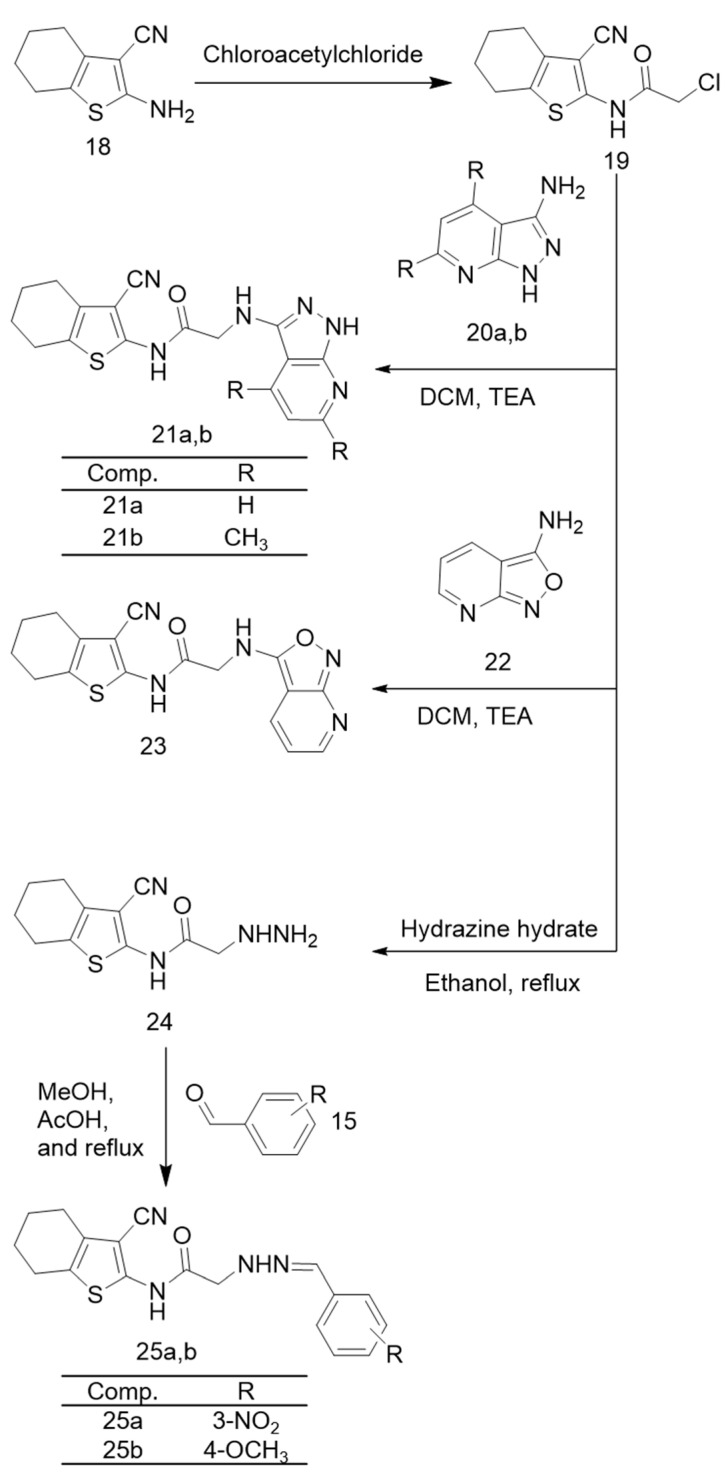
Synthesis of tetrahydrobenzothieno-acetamide derivatives (series C).

The synthetic methods for series compounds A/B and C were illustrated in Scheme 1 and Scheme 2. Compounds (**11**–**13**) were synthesized as reported in our earlier research [16]. Series A and B compounds were obtained by condensation of compounds (**13a**,**b**) with different aromatic aldehydes (Scheme 1) [17]. Scheme 2, involving the synthesis of series C, started with the reaction of 2-amino-5,6-dihydro-4H-cyclohexa[b]thiophene-3-carbonitrile (**18**) with chloroacetyl chloride to yield compound (**19**). Then, coupling of compound (**19**) with heteroaryl amines (**20a**,**b** and **22**) [18] and hydrazine hydrate gave compounds (**21a**,**b**), (**23**), and (**24**) respectively [19]. The hydrazide derivative (**24**) was condensed with different aromatic aldehydes, yielding compounds (**25a**,**b**) [17].

The structures of the synthesized compounds were confirmed by microanalyses and spectral data (IR, ^1^H NMR, and EI-MS). Mass spectra showed the expected molecular ion peaks. IR spectra of the synthesized compounds (**21a**,**b**) collectively showed secondary amine signals at around 3300–3500 cm^−1^, which indicates coupling between 2-chloro-*N*-(3-cyano-4,5,6,7-tetrahydrobenzo[b] thiophen-2-yl)acetamide (**19**) and heteroaryl amines (**20a**,**b** and **22**) with the disappearance of signal corresponding to NH_2_. ^1^H NMR spectra of compounds (**14**–**17a**) showed multiple peaks of aromatic protons in the range of 7.80 to 8.70 δ (ppm) and a single peak for CH proton in the range of 7.4 to 8.4 δ (ppm). The spectrum of compound (**15a**) showed a single peak corresponding to OH proton at 10.15 δ (ppm), and the spectrum of compound (**16a**) showed a single sharp peak at 4.06 δ (ppm) corresponding to the OCH_3_ group of anisaldehyde. A single peak for CH proton appeared at 7.85 δ (ppm) and 7.64 δ (ppm) for compounds (**15b**) and (**17b**), respectively. The ^1^H NMR spectrum of compound (**15b**) showed a single peak corresponding to OH proton at 8.25 δ (ppm). The ^1^H NMR spectrum of compound (**21a**) showed the appearance of single peak at 11.88 δ (ppm) and multiple peaks at 6.95 δ (ppm) corresponding to NH proton and three aromatic protons of heteroarylamine, respectively. The NH proton of the coupling reaction appeared as a single peak at 3.65 δ (ppm). Another single peak belonging to proton of NH of thiophene appeared at 5.49 δ (ppm). Finally, a single sharp peak accounting for CH_2_ appeared at 3.08 δ (ppm). The ^1^H NMR spectrum of compound (**21b**) showed the appearance of single peaks at 11.69 and 6.58 δ (ppm), corresponding to NH proton and single aromatic proton of the heteroaryl amine, respectively. A single peak for NH proton of thiophene appeared at 5.01 δ (ppm) and a sharp peak of CH_2_ appeared at 3.65 δ (ppm). The spectrum also showed two single sharp peaks for the two methyl groups of the heteroarylamine at 2.42 and 2.54 δ (ppm).

### 2.2. Biological Evaluation

#### 2.2.1. In Vitro Cytotoxic Activity against H1299 Cell Line

**Figure 3:**
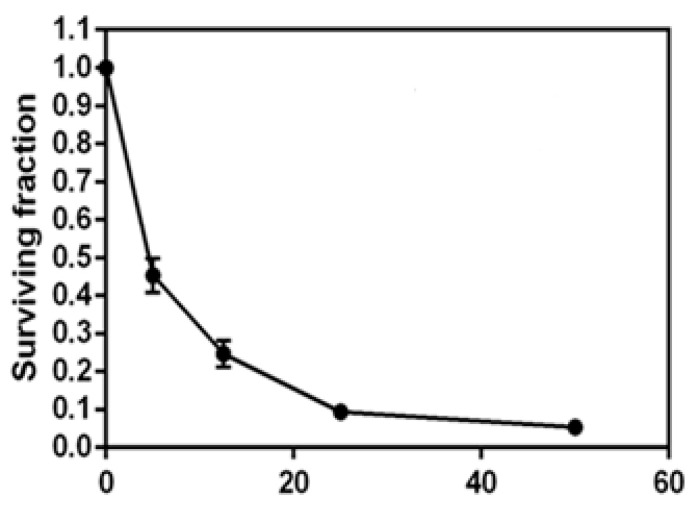
Viability curve of compound (**21a**), with cytotoxic activity against H1299 NSCLC cell line.

The in vitro cytotoxic activities of the synthesized compounds (**13**–**25a**,**b**) were measured using sulfo-rhodamine B (SRB) assay [20] against the NSCLC cell line H1299 and gefitinib (**2**) as a reference drug. This cancer cell line has high expression of EGFR and HER2 enzymes [3]. As seen in Table 1, the results revealed that the synthesized compounds showed versatile antitumor activity against the tested cell line. The synthesized compounds (**13**–**25a**,**b**) showed IC_50_ values in the range of 12 to 54 nM, superior to that of gefitinib (**2**) at 40 µM. Compound (**21a**) was the most potent, with IC_50_ = 12.5 nM, followed by compound (**21b**), with IC_50_ = 13.68 nM. The viability curve of compound (**21a**), with the most potent anticancer activity, is displayed in Figure 3.

#### 2.2.2. EGFR and HER2 Kinase Inhibitory Assay

The most active compound (**21a**) was chosen for further evaluation of in vitro EGFR and HER2 kinase inhibitory activity. The results showed that the IC_50_ values of compound (**21a**) were 0.47 and 0.14 nM against EGFR and HER2, respectively. This is superior to the value of gefitinib (**2**) against EGFR (1.9 nM). It is comparable to IC_50_ values for imatinib (**1**) 0.11 nM against EGFR and 0.06 nM against HER2 (Table 2).

### 2.3. Molecular Modeling

#### 2.3.1. Molecular Docking

**Figure 4:**
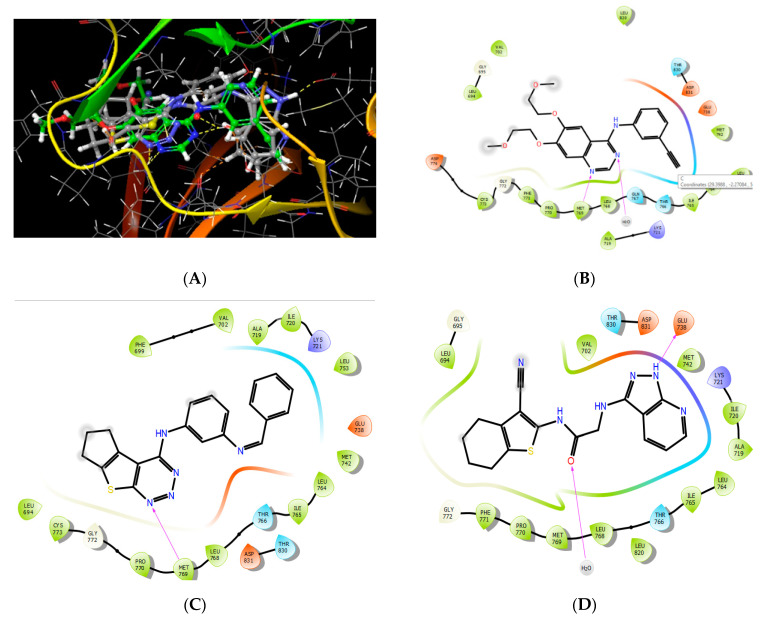
(**A**) Three-dimensional binding modes and overlay of erlotinib (**3**), compound (**14a**), and compound (**21a**) in the catalytic domain of EGFR (PDB 1M17). (**B**) 2D representation of erlotinib (**3**) in the EGFR catalytic domain. (**C**) 2D representation of compound (**14a**) in the EGFR catalytic domain. (**D**) 2D representation of compound (**21a**) in the EGFR catalytic domain.

Computer-aided simulation of drug design saves effort, time, and energy compared to conventional methods of drug production. Molecular-based drug design was proposed in order to predict the possible interactions of the target compounds with EGFR/HER2. Using Glide in the Schrodinger program for docking, the modulation activity of erlotinib (**3**) and afatinib (**5**) derivatives against the EGFR/HER2 receptor was determined, as shown in Figure 4. The designed compounds were docked into the ATP-binding pocket as a thienotriazine and tetrahydrobenzothiophene scaffold, mimicking the ATP adenine ring occupying the hinge region motif of TKs. The positions of thienotriazine (**14a**), tetrahydrobenzothiophene (**21a**), erlotinib (**3**), and afatinib (**5**) were similar, which implies that they might share the same biological activity. The docking results show, in detail, that the designed TKI formed hydrogen bonds, hydrophobic contacts, and hydrophobic–hydrophilic interactions with the target TKs. N1 of the triazine motif in series A and B could act as a hydrogen bond acceptor and form hydrogen bonds with MET769 (distance 2.4 Å, angle 143.6) in the hinge region, mimicking the N1 of quinazoline in the approved drug erlotinib (**3**) (distance 2.2 A, angle 135.7). In series C, a tetrahydrobenzothiophene scaffold occupied the hinge region in the same position of quinazoline, forming a hydrogen bond with crystal water, mimicking the N3 of erlotinib’s quinazoline. The phenyleneimine and pyrazolopyridine moieties deeply penetrated the hydrophobic pocket, forming hydrophobic–hydrophobic, Van der Waals, and π–π interactions with key amino acids such as ALA719, ILE720, MET742, LEU753, and LEU764, mimicking the phenylacetylene of erlotinib (**3**). Modifying these moieties, such as by adding hydroxy, nitro, or methoxy groups, could enhance affinity through the formation of hydrogen bonds with GLU738 and LYS721. The incorporation of CN at position 3 of tetrahydrobenzothiophene could confer series C with a certain superiority by forming hydrophobic interactions with LEU694 and VAL702. Extra precision glide docking of compounds (**14a**) and (**21a**) with the active domain of EGFR showed reasonable docking scores of −6.78 and −7.69 and glide E-model values of −64.6 and −71.2 kcal mol^−1^, respectively.

**Figure 5:**
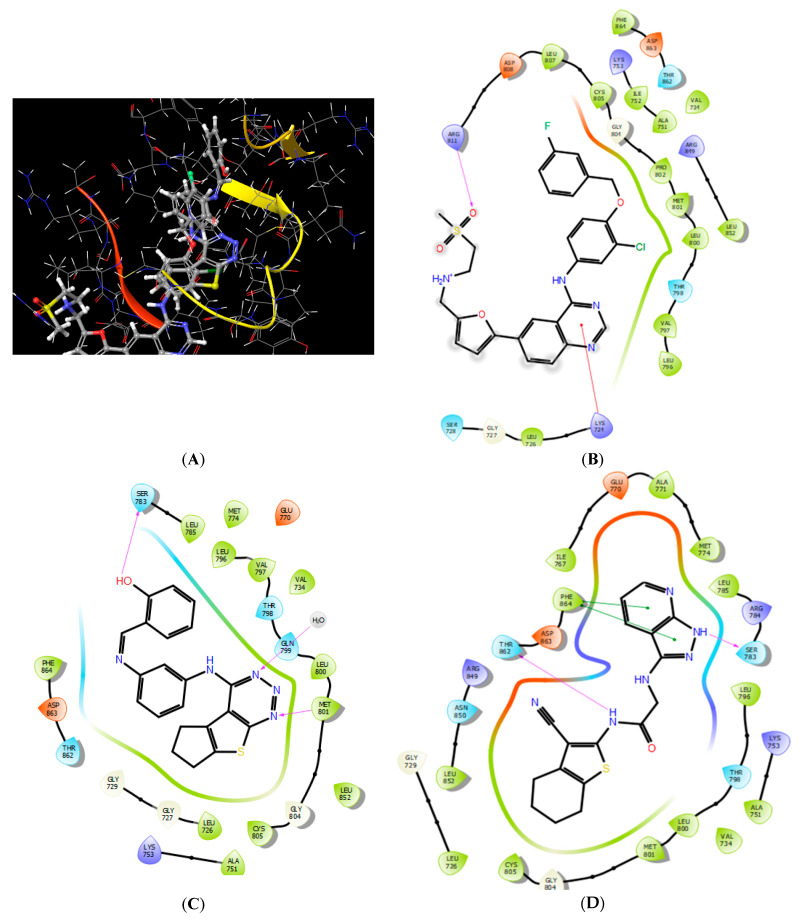
(**A**) Three-dimensional binding modes and overlay of lapatinib (**4**), compound (**15a**), and compound (**21a**) in the catalytic domain of HER2 (PDB 3PP0). (**B**) 2D representation of lapatinib (**4**) in the catalytic domain of HER2. (**C**) 2D representation of compound (**15a**) in the catalytic domain of HER2. (**D**) 2D representation of compound (**21a**) in the catalytic domain of HER2.

On the other hand, the docking study of the designed compounds in human HER2 showed high affinity of these compounds toward this receptor. Thienotriazine and tetrahydrobenzothiophene occupied the hinge region, forming hydrogen bonds with the MET803 and water molecules there, as shown in Figure 5. Additionally, the hydrophobic heads in the upper right position of series A and B and at position 2 of series C deeply penetrated the hydrophobic pocket in HER2, forming hydrogen bond, hydrophobic, Van der Waals, and π–π interactions. The inclusion of large substitutions was intended to improve the affinity of the designed compounds toward HER2, generating dual-acting compounds mimicking lapatinib (**4**) and neratinib (**6**). The extra precision docking score and glide energy showed the highest affinity of the design compounds compared to lapatinib (**4**) and neratinib (**6**). For (**15a**) and (**21a**), the docking scores were −10.5 and −8.8, and the glide energy values were −81.4 and −77.03 kcal mol^−1^, respectively. These values are higher than those of lapatinib (**4**) (−7.8 and −79.8 kcal mol^−1^) and neratinib (**6**) (−4.3 and −52.6 kcal mol^−1^). These results are in agreement with those of in situ and in vitro screening studies.

**Figure 6:**
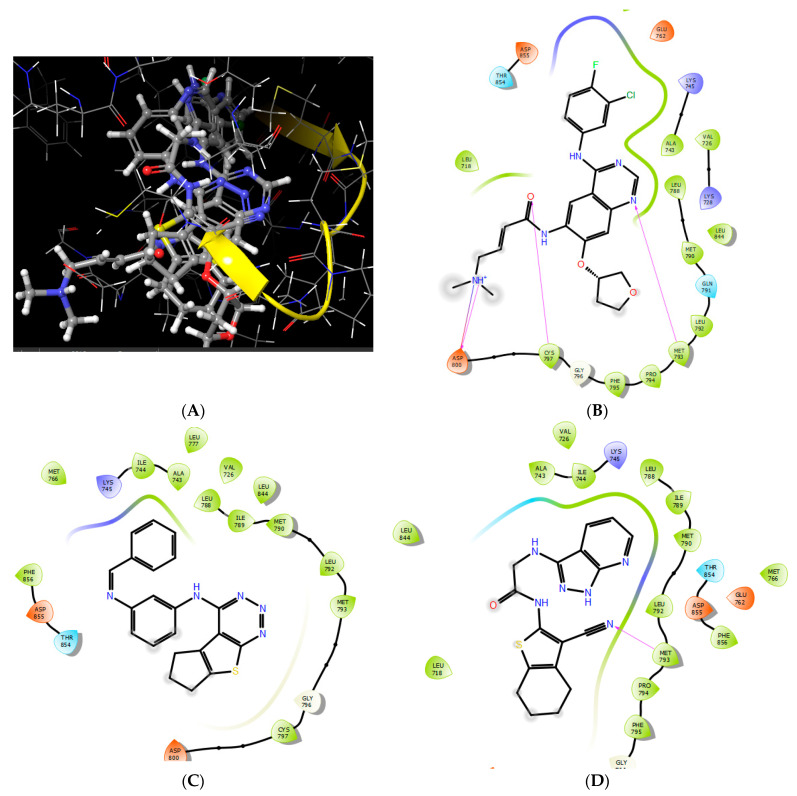
(**A**) Three-dimensional binding modes and overlay of afatinib (**5**) and compounds (**14a**) and (**21a**) in the catalytic domain of T790M EGFR (PDB 4G5P). (**B**) 2D representation of afatinib (**5**) in the catalytic domain of T790M EGFR. (**C**) 2D representation of compound (**14a**) in the catalytic domain of T790M EGFR. (**D**) 2D representation of compound (**21a**) in the catalytic domain of T790M EGFR.

Acquired drug resistance normally develops after about 12 months of treatment with gefitinib (**2**) or erlotinib (**3**), which is attributed to secondary T790M mutation. To confirm the benefits of the designed compounds in terms of their affinity toward mutated TKs, the molecular docking study was extended to include mutated T790M protein (PDB 4G5P), as shown in Figure 6. The designed compounds occupied the same position as afatinib (**5**). The amide link at position 2 of the thiophene ring and the cyano group at position 3 of the highest potent compound (**21a**) formed a hydrogen bond (2.01 A, angle 108.9) with MET793 in the hinge region, mimicking the hydrogen bond of N1 of the quinazoline ring in afatinib (**5**) (2.19 Å, angle 116.8). Pyrazolopyridine participated in hydrophobic interactions, enforcing the binding affinity. The electrophilic moiety and hydrophobic tail at the left side of quinazoline formed extra hydrogen bonds, hydrophobic interactions, and covalent bonds with CYS797. Extra precision glide docking of compounds (**14a**) and (**21a**) with the active domain of T790M EGFR showed reasonable docking scores of −7.7 and −7.5 and glide E-model values of −72.12 and −66.88 kcal mol^−1^, respectively. These values were not as high as to those of afatinib (**5**) (−10.5 and −103.4 kcal mol^−1^); however, they might better than those of lapatinib (**4**) (−6.2 and −87.2 kcal mol^−1^).

#### 2.3.2. Physicochemical Properties and In Silico ADME Prediction

The pharmacokinetic properties of drug-like molecules are primarily determined by the physicochemical properties of the designed molecules, such as solubility in aqueous solution, lipophilicity, hydrogen bonding capability, polar surface area, chemical stability, etc. Significantly, the physiochemical properties of a compound are the key to turning a biologically active compound into a therapeutically effective drug [16]. Therefore, our promising novel compound (**21a**) and moderately active compound (**15a**) were subjected to prediction of physicochemical properties using ADME predictions made by the QikProp v4.3 function of Schrodinger 10-1 prior to in vivo studies, with the licensed drug lapatinib (**4**) used as a reference compound. According to the QikProp suite, polar surface area (PSA), partition coefficient (log PO/W), and number of rotatable bonds were predicted in order to test the possible drug-like properties of compounds. As seen in Table 3, compounds (**15a**) and (**21a**) exhibited appropriate physicochemical properties compared to lapatinib (**4**). Caco-2 cell permeability (QPPCaco) was used as a marker for intestinal absorption or permeation. Compound (**15a**) showed a high partition coefficient and, consequently, the highest cell permeability. The most active compound (**21a**) and compound (**15a**) might be considered as promising lead molecules for designing potent EGFR inhibitors with excellent membrane permeability and oral bioavailability.

#### 2.3.3. Molecular Orbital Energy Study and Molecular Electrostatic Potential (MESP)

**Figure 7:**
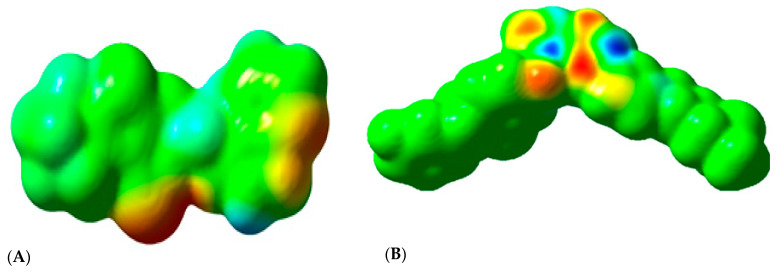
Molecular electrostatic potential mapping for (**A**) the most potent compound (**21a**) and (**B**) lapatinib (**4**) from density functional theory (DFT) calculations. Green indicates zero potential regions, blue indicates electropositive regions, and red indicates electronegative regions.

A molecular electrostatic potential (MESP) map is a useful tool for understanding the sites for nucleophilic reactions, electrophilic attacks, and intermolecular interactions. Additionally, it widely used for the detection of iso-surface values, showing the locations of positive and negative electrostatic potential of designed compounds [16]. The MESP values of compounds (**21a**) and lapatinib (**4**) are shown in Figure 7. Red indicates the most electronegative region, blue the most electropositive region, and green the region of zero potential. The nitrogen atoms of quinazoline and aniline and the oxygen group of the furan ring of lapatinib (**4**) have a greater negative charge surrounded by some carbon atoms with greater positive charge. Thus, electrophilic attack and nucleophilic interaction with the receptor are more favorable for these parts of lapatinib (**4**). The remaining parts of the structure are shown in green, which means they can bind through hydrophobic and Van der Waals interactions. In the same manner, the electronegative and electropositive regions of the designed compounds are located around 3-cyanotetrahydrothiophen and pyrazolopyridine, making these regions better able to interact with charged parts of the EGFR/HER2 receptors. The electronegative region around pyrazolopyridine distinguished it somewhat from lapatinib (**4**), which lacks this. The docking study showed that the designed compounds have higher affinity toward EGFR/HER2 compared to lapatinib (**4**). Therefore, we attributed the high affinity and biological activity observed in the in vitro study to the inclusion of a heterocyclic rather than aromatic system in this region that is common in TKIs.

**Figure 8:**
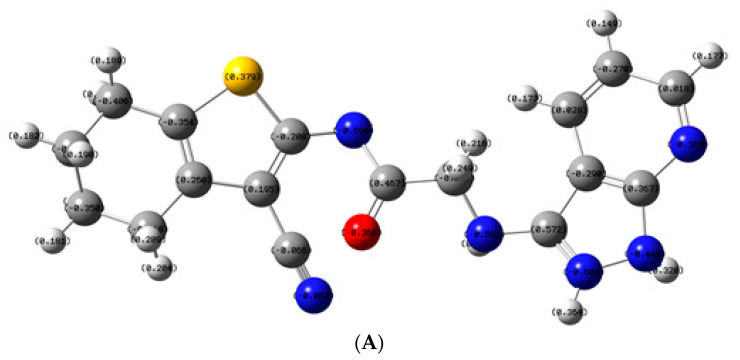

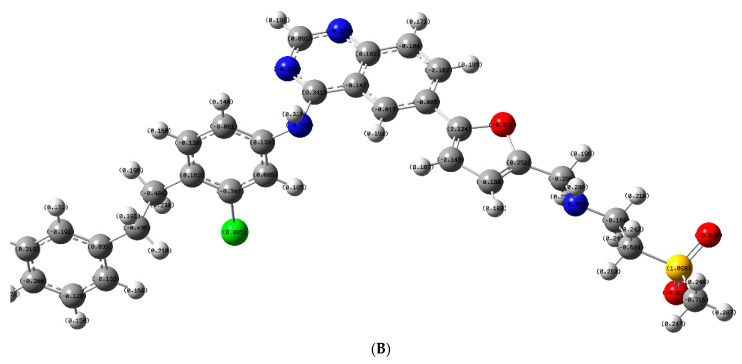
Mulliken atomic charges for (**A**) the most potent compound (**21a**) and (**B**) lapatinib (**4**).

Moreover, the Mulliken atomic charges (Figure 8) are in agreement with the MESP results. The negative charge is mainly located on nitrogen atoms of the quinazoline of lapatinib (**4**) and on the cyano group and nitrogen of the amide link and nitrogen atoms of pyrazolopyridine in the most potent compound (**21a**). The highest negative charge in both lapatinib (**4**) and the representative compound give this atom the highest reactivity in the interaction with EGFR and HER2 receptors.

**Figure 9:**
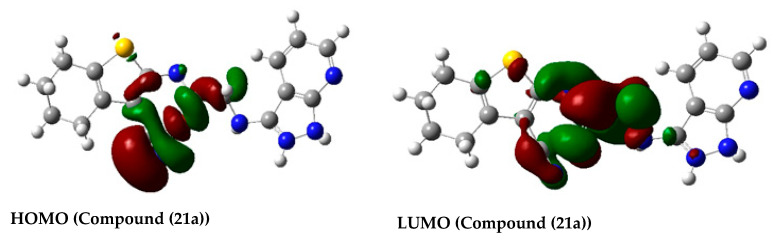

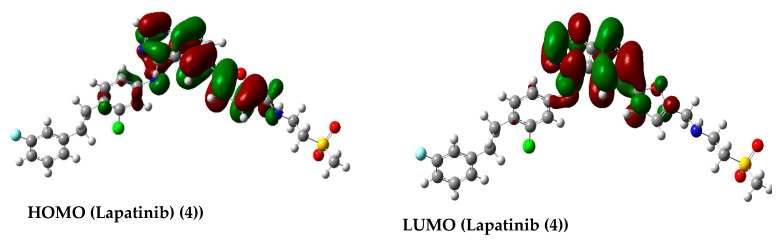
Highest occupied molecular orbital (HOMO) and lowest unoccupied molecular orbital (LUMO) maps for compound (**21a**) and lapatinib (**4**) from DFT calculations. Green indicates positive molecular orbital and red indicates negative molecular orbital.

The highest occupied molecular orbital (HOMO) and lowest unoccupied molecular orbital (LUMO) maps of the representative compound (**21a**) and lapatinib (**4**) are shown in Figure 9. On the one hand, it was observed that the electrons were delocalized on quinazoline, aniline, furan, and amino moieties in the HOMO map, whereas on the LUMO map, the electrons are mainly delocalized on quinazoline and aniline groups. On the other hand, the HOMO and LUMO maps of the target compounds are delocalized around the 3-cyanothiophene and amide linkage. The frontier molecular orbitals are mainly delocalized on the moieties or groups that mainly participate in drug–receptor interaction. Therefore, we can conclude that the biological activity might be facilitated via the quinazoline moiety of lapatinib (**4**) and the 3-cyanothiophene moiety of the designed compound through hydrophobic, hydrophilic, Van der Waals, and π–π interactions or via hydrogen bond formation with key amino acids in the catalytic domain. The LUMO map and part of the HOMO map of the designed compounds and lapatinib (**4**) are concentrated on 3-cyanothiophene and quinazoline, making these moieties superior in drug–receptor interaction. Besides this, the amide linkage also participated in this interaction. The results of density functional theory (DFT) study are in accordance with those of the docking and in vitro cytotoxicity studies. Gaussian 09 software (Gauss View 5.0, Gaussian, Inc., Wallingford, CT, USA) was utilized for all computational calculation.

TK proteins are ATP-dependent, and the ATP-binding domain is conserved in TKs and serves as the receptor for anticancer agents. The discovery of novel inhibitors is focused mainly on searching for compounds containing the proper chemical moieties that can fulfill the requirements for interaction with the adenine-binding site (hinge region); 4-anilino-quinoline and/or quinazoline are features common in several approved TKIs. However, it was found that for most of the quinazoline-based anticancer agents that selectively inhibit EGFR/HER2 overexpression, resistance is developed over long courses of treatment. The discovery of a novel core structure with various substitutions at different positions is thus of great importance.

**Figure 10:**
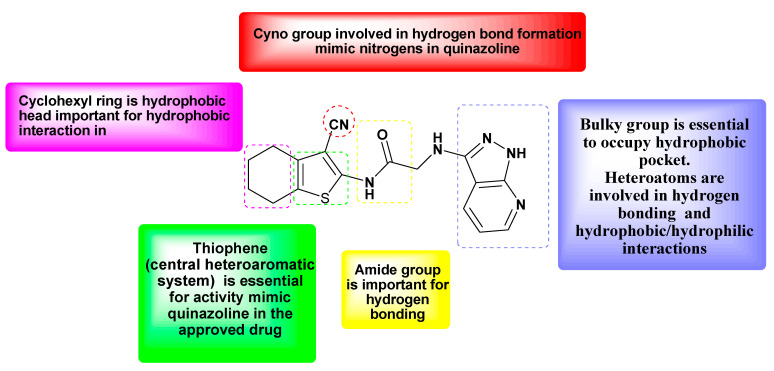
The observed structure–activity relationships of the designed molecules as promising dual EGFR/HER2 inhibitors.

In this study, and based on our previous publication, thienotriazine and 3-cyanotetrahydrobenzothiophene were selected as the core scaffold to design novel dual-acting EGFR/HER2 drug-like molecules. The designed compounds displayed promising in situ cytotoxicity against H1299 NSCLC and in vitro activity against EGFR and HER2, comparable to the approved TKIs imatinib (**1**) and gefitinib (**2**). The molecular modeling along with the computational studies revealed the significance of the two core moieties—thienotriazine and 3-cyanotetrahydrobenzothiophene, which functioned as the adenine of ATP in the hinge region, forming hydrogen bonding interactions. Modifying the upper right hydrophobic head through the addition of some heterocyclic systems (pyrazolopyridine and isoxazolopyridine) resulted in superior activity of the designed compound compared with the approved dual-acting drug lapatinib (**4**). Heterocyclic systems succeeded in forming extra hydrogen bonding in addition to the hydrophobic interaction in the hydrophobic region. The red electronic map of MESP and the highly negative charge localized in these systems, shown by Mulliken charge distribution, potentiated the merit of these moieties. Furthermore, alteration in the position of the substitution from position 4 on quinazoline in the common approved TKI drugs to position 2 in our series C enforced the potency and conferred to the designed compounds a certain benefit in strongly interacting with TKs at different sites. Molecular modeling also revealed that these modifications increased the interaction of the designed compounds with the protein kinase harboring the T790M mutation, which is responsible for acquired drug resistance. Figure 10 can summarize the cytotoxic activity of the designed molecules based on the observed structure–activity relationships (SAR).

## 3. Materials and Methods

### 3.1. Instrument

The Varian Mercury Vx 300 NMR spectrometer or JEOL LA (300 MHz for ^1^H NMR) were used to measure ^1^H NMR spectra. Electron impact mass spectra (EI-MS) were recorded on a Shimadzu GCMS-QP 5050A gas chromatograph mass spectrometer (70 eV). Melting points were calculated in open capillaries using a Gallenkemp melting point apparatus and are uncorrected. Infrared (IR) spectra were recorded on a Shimadzu FT-IR 8101 PC IR spectrophotometer (KBr pellets).

### 3.2. Chemicals and Reagents

Commercial chemicals and solvents of reagent grade were used directly in synthesis without further purification.

### 3.3. Experimental

#### Chemistry

**General procedure for synthesis of compounds (14a–17a)**

A mixture of 3-(5,6-dihydro-7*H*-cyclopenta[4:5]thieno[2,3-d][1,2,3]triazin-4-ylamino)benzene-1,3-diamine (**13a**) [16] (0.01 mol) and suitable aromatic aldehyde (0.01 mol) in methanol (20 mL) in the presence of a catalytic amount of glacial acetic acid was refluxed for 5 h, with the progression of reaction monitored using TLC. The solvent was removed under reduced pressure, and the product recrystallized from chloroform [17].

**3-(5,6-Dihydro-7*H*-cyclopenta[4:5]thieno[2,3-d][1,2,3]triazin-4-ylamino)-*N*-benzylidenebenzene-1,3-diamine (14a)**

Mass spectrum: *m*/*z* (%): 371 (M^+^, 1.50%), 361 (1.33%), 353 (1.21%), 343 (7.82%), 316 (11.55%), 108 (100%). IR (cm^−1^): 752 (C–S), 1261 (C–N), 1624 (C=N), 3429 (N–H). ^1^H NMR (DMSO, 300 MHz): *δ* (ppm) 1.29 (m, 2H), 3.08 (t, 2H), 3.27 (t, 2H), 7.08 (m, 8H), 7.71 (s, 1H), 7.80 (s, 1H), 11.81 (s, 1H). Elemental analysis calculated for C_21_H_17_N_5_S: C, 67.90; H, 4.61; N, 18.85. Found: C, 68.09; H, 4.66; N, 19.08.

**3-(5,6-Dihydro-7*H*-cyclopenta[4:5]thieno[2,3-d][1,2,3]triazin-4-ylamino)-*N*-(2-hydroxybenzylidene)benzene-1,3-diamine (15a)**

Mass spectrum: *m/z* (%): 387 (M+, 0.76%), 375 (2.45%), 347 (6.59%), 345 (9.14%), 212 (85.96%), 119 (100%). IR (cm^−1^): 756 (C–S), 1149 (C–O), 1276 (C–N), 1616 (C=N), 3221 (N–H), 3352 (OH). ^1^H NMR (DMSO, 300 MHz): *δ* (ppm) 1.14 (m, 2H), 2.12 (t, 2H), 2.73 (t, 2H), 4.54 (s, 1H), 7.84 (m, 7H), 7.90 (s, 1H), 8.52 (s, 1H), 10.15 (s, 1H). Elemental analysis calculated for C_21_H_17_N_5_OS: C, 65.10; H, 4.42; N, 18.08. Found: C, 65.34; H, 4.47; N, 18.39.

**3-(5,6-Dihydro-7*H*-cyclopenta[4:5]thieno[2,3-d][1,2,3]triazin-4-ylamino)-*N*-(3-nitrobenzylidene)benzene-1,3-diamine (16a)**

Mass spectrum: *m/z* (%): 401 (M^+^, 3.10%), 389 (79.4%), 362 (1.80%), 322 (1.20%), 293 (2.40%), 261 (1.50%), 249 (100%). IR (cm^−1^): 690 (C–S), 1246 (C–O–C), 1261 (C–N), 1678 (C=N), 3444 (N–H). ^1^H NMR (DMSO, 300 MHz): *δ* (ppm): 1.15 (m, 2H), 3.65 (t, 2H), 3.70 (t, 2H), 4.06 (s, 3H), 7.30 (m, 7H), 7.85 (s, 1H), 8.70 (s, 1H), 10.20 (s, 1H). Elemental analysis calculated for C_22_H_19_N_5_OS: C, 65.81; H, 4.77; N, 17.44. Found: C, 66.04; H, 4.84; N, 17.68.

**3-(5,6-Dihydro-7*H*-cyclopenta[4:5]thieno[2,3-d][1,2,3]triazin-4-ylamino)-*N*-(4-methoxybenzylidene)benzene-1,3- diamine (17a)**

Mass spectrum: *m/z* (%): 416 (M^+^, 0.88%), 407 (2.47%), 403 (2.56%), 376 (4.56%), 222 (12.24%), 108 (100%). IR (cm^−1^): 732 (C–S), 1350 (NO_2_), 1527 (NO_2_), 1612 (C=N), 3363 (N–H). ^1^H NMR (DMSO, 300 MHz): *δ* (ppm): 1.80 (m, 2H), 2.62 (t, 2H), 2.64 (t, 2H), 4.80 (s, 1H), 7.28 (m, 6H), 7.31 (s, 1H), 7.34 (s, 1H), 8.50 (s, 1H). Elemental analysis calculated for C_21_H_16_N_6_O_2_S: C, 60.56; H, 3.87; N, 20.18. Found: C, 60.73; H, 3.91; N, 20.43.

**General procedure for synthesis of compounds (15b) and (17b)**

A mixture of 3-(5,6,7,8-Tetrahydro-7*H*-cyclohexa[4:5]thieno[2,3-d][1,2,3]triazin-4ylamino)benzene-1,3-diamine (**13b**) [16] (0.01 mol) and suitable aromatic aldehyde (0.01 mol) in methanol (20 mL) in the presence of a catalytic amount of glacial acetic acid was refluxed for 5 h, with the progression of reaction monitored using TLC. The solvent was removed under reduced pressure, and the product recrystallized from chloroform [17].

**3-(5,6,7,8-Tetrahydro-7*H*-cyclohexa[4:5]thieno[2,3-d][1,2,3]triazin-4-ylamino)-*N*-(2-hydroxybenzylidene)benzene-1,3-diamine (15b)**

Mass spectrum: *m/z* (%): 401 (M^+^, 7.20%), 390 (3.80%), 375 (2.10%), 361 (6.49%), 346 (100%), 303 (5.30%). IR (cm^−1^): 756 (C–S), 1678 (C=N), 3228 (N–H), 3363 (OH). ^1^H NMR (DMSO, 300 MHz): *δ* (ppm): 1.13 (m, 4H), 3.05 (t, 2H), 3.10 (t, 2H), 3.60 (s, 1H), 7.13 (m, 7H), 7.81 (s, 1H), 7.85 (s, 1H), 8.25 (s, 1H). Elemental analysis calculated for C_22_H_19_N_5_OS: C, 65.81; H, 4.77; N, 17.44. Found C, 65.58; H, 4.39; N, 17.76.

**3-(5,6,7,8-Tetrahydro-7*H*-cyclohexa[4:5]thieno[2,3-d][1,2,3]triazin-4-ylamino)-*N*-(3-nitrobenzylidene)benzene-1,3- diamine (17b)**

Mass spectrum: *m/z* (%): 430 (M^+^, 5.10%), 384 (8.60%), 354 (10.40%), 296 (6.30%), 103 (100%). IR (cm^−1^): 732 (C–S), 1350 (NO_2_), 1527 (NO_2_), 3360 (N–H). ^1^H NMR (DMSO, 300 MHz): *δ* (ppm): 1.14 (m, 4H), 3.04 (t, 2H), 3.11 (t, 2H), 3.20 (s, 1H), 7.31 (m, 6H), 7.37 (s, 1H), 7.45 (s, 1H), 7.64 (s, 1H).

**2-Chloro-*N*-(3-cyano-4,5,6,7-tetrahydrobenzo[b]thiophen-2-yl)acetamide (19)**

To a stirred solution of 2-amino-4,5,6,7-tetrahydrobenzo[b]thiophene-3-carbonitrile (**11**) [22] (0.10 mol) in acetone (20 mL), chloroacetyl chloride (0.05 mol) at 0–5 °C was added dropwise and maintained in an ice bath. The reaction mixture was stirred at room temperature for an additional 4 h. Then, 40 mL of 10% HCl was added to the mixture. The formed precipitate was filtered and washed with HCl (10%) and water. Melting point: 175–177 °C [23].

**General procedure for synthesis of compounds (21a,b)**

The amine derivatives (**20a,b**) (0.009 mol) [24] were dissolved in dry dichloromethane (DCM) (25.0 mL) and triethylamine (TEA) (1.5 mL) under nitrogen atmosphere. Compound (19) (0.0045 mol) was slowly added to the stirred solution, and the solution was then refluxed for 6–9 h. The mixture was then cooled and poured onto crushed ice. The formed precipitate was filtered, washed with water, dried and recrystallized from absolute ethanol [4].

**2-(1*H*-Pyrazolo[3,4-b]pyridin-3-ylamino)-*N*-(3-cyano4,5,6,7-tetrahydrobenzo[b]thiophen-2-yl)acetamide (21a)**

Mass spectrum: *m/z* (%): 354 (M^+2^, 4.29%), 353 (4.83%), 352 (6.96%), 320 (57.61%), 291 (38.64%), 178 (100%). IR (cm^−1^): 789 (C–S), 1689 (C=O), 2224 (C≡N), 3214 (N–H), 3338 (N–H), 3443 (N–H). ^1^H NMR (DMSO, 300 MHz): *δ* (ppm): 1.20 (m, 4H), 3.06 (m, 4H), 3.08 (s, 2H), 3.65 (s, 1H), 5.49 (s, 1H), 6.95 (m, 3H), 11.88 (s, 1H). Elemental analysis calculated for C_17_H_16_N_6_OS: C, 57.94; H, 4.58; N, 23.85. Found: C, 58.21; H, 4.42; N, 24.18.

***N*-(3-Cyano-4,5,6,7-tetrahydrobenzo[b]thiophen-2-yl)-2-(4,6-dimethyl-1*H*-pyrazolo[3,4-b]pyridin-3-ylamino)acetamide (21b)**

Mass spectrum: *m/z* (%) 382 (M^+2^, 1.22%), 381 (1.08%), 380 (3.65%), 348 (1.44%), 320 (13.61%), 143 (100%). IR (cm^−1^): 778 (C–S), 1689 (C=O), 2224 (C≡N), 3285 (N–H), 3388 (N–H), 3444 (N–H). ^1^H NMR (DMSO, 300 MHz): *δ* (ppm): 1.18 (m, 4H), 1.75 (s, 1H), 2.42 (s, 3H), 2.54 (s, 3H), 3.02 (m, 4H), 3.65 (s, 2H), 5.01 (s, 1H), 6.58 (s, 1H), 11.69 (s, 1H). Elemental analysis calculated for C_19_H_20_N_6_OS: C, 59.98; H, 5.30; N, 22.09. Found: C, 60.29; H, 5.43; N, 22.41.

***N*-(3-Cyano-4,5,6,7-tetrahydrobenzo[b]thiophen-2-yl)-2-(isoxazolo[3,4-b]pyridin-3-ylamino)acetamide (23)**

Compound (**22**) [24] was dissolved in dry DCM (25.0 mL) and TEA (1.5 mL) under nitrogen atmosphere. Compound (19) (0.0045 mol) was added slowly to the stirred solution, then heated under reflux for 6–9 h. The mixture was then cooled and poured onto crushed ice. The formed precipitate was filtered and washed with water, dried and recrystallized from absolute ethanol [4].

Mass spectrum: *m/z* (%): 355 (M^+2^, 0.39%), 354 (1.95%), 353 (0.60%), 343 (4.50%), 313 (6.29%), 144 (100%). IR (cm^−1^): 692 (C–S), 1689 (C=O), 2224 (C≡N), 3220 (N–H), 3446 (N–H). ^1^H NMR (DMSO, 300 MHz): *δ* (ppm): 1.79 (m, 4H), 3.17 (m, 4H), 3.82 (s, 2H), 4.09 (s, 1H), 7.09 (m, 3H), 9.78 (s, 1H). Elemental analysis calculated for C_17_H_15_N_5_O_2_S: C, 57.78; H, 4.28; N, 19.82. Found: C, 58.01; H, 4.37; N, 20.09.

***N*-(3-Cyano-4, 5, 6, 7-tetrahydrobenzo[b]thiophen-2-yl)-2-hydrazinyl acetamide (24)**

A mixture of hydrazine hydrate 80% (0.05 mol, 0.25 mL) and 2-chloro-*N*-(3-cyano-4,5,6,7-tetrahydrobenzo[b]thiophen-2-yl)acetamide (**19**) (0.0015 mol) in absolute ethanol (20 mL) was refluxed for 4 h, with the progression of reaction monitored using TLC. The reaction mixture was cooled and poured into water. The formed precipitate was filtered and recrystallized from ethanol to give the desired hydrazide. Melting point: 140–142 °C [19].

**General procedure for synthesis of compounds (25a,b)**

A mixture of compound (**24**) (0.01 mol) and the suitable aromatic aldehyde (0.01 mol) dissolved in methanol (20 mL) in the presence of a catalytic amount of glacial acetic acid was refluxed for 5 h, with the progression of reaction monitored using TLC. The solvent was removed under reduced pressure, and the product recrystallized from chloroform [17].

***N*-(3-Cyano-4,5,6,7-tetrahydrobenzo[b]thiophen-2-yl)-2-(2-(3-nitrobenzylidene)hydrazinyl)acetamide (25a)**

Mass spectrum: *m/z* (%) 385 (M^+2^, 0.66%), 384 (1.26%), 383 (2.27%), 369 (16.60%), 339 (5.59%), 311 (100%). IR (cm^−1^): 736 (C–S), 1265 (NO_2_), 1526 (NO_2_), 1689 (C=O), 2221 (C≡N), 3088 (N–H), 3443 (N–H). ^1^H NMR (DMSO, 300 MHz): *δ* (ppm): 1.78 (m, 4H), 2.15 (s, 1H), 2.45 (m, 4H), 3.62 (s, 2H), 7.80 (m, 3H), 8.71 (s, 1H), 8.88 (s, 1H), 11.63 (s, 1H). Elemental analysis calculated for C_18_H_17_N_5_O_3_S: C, 56.38; H, 4.47; N, 18.27. Found: C, 56.70; H, 4.58; N, 18.49.

***N*-(3-Cyano-4,5,6,7-tetrahydrobenzo[b]thiophen-2-yl)-2-(2-(4-methoxybenzylidene)hydrazinyl)acetamide (25b)**

Mass spectrum: *m/z* (%) 370 (M^+2^, 2.36%), 369 (6.14%), 368 (21.44%), 354 (100%), 338 (5.20%), 296 (39.14%). IR (cm^−1^): 695 (C–S), 1256 (OCH_3_), 1689 (C=O), 2225 (C≡N), 3220 (N–H), 3446 (N–H). ^1^H NMR (DMSO, 300 MHz): *δ* (ppm): 1.74 (m, 4H), 2.16 (s, 1H), 2.56 (m, 4H), 3.63 (s, 2H), 3.82 (s, 3H), 7.04 (m, 4H), 8.62 (s, 1H), 11.64 (s, 1H). Elemental analysis calculated for C_19_H_20_N_4_O_2_S: C, 61.94; H, 5.47; N, 15.21. Found: C, 62.23; H, 5.60; N, 15.49.

### 3.4. Biological Assays

#### 3.4.1. Cytotoxic Activity against H1299

The potential cytotoxic activity of the target compounds on H1299 lung cancer cell line was tested using sulfo-rhodamine B (SRB) assay according to Skehan method [20]. Single cells were plated in 96-multiwell plates for 24 h before treatment with the compounds. A single concentration of the tested compounds was added to the cell monolayer. Monolayer cell was incubated with the compounds for 48 h at 37 °C and in atmosphere of 5% CO_2_. After 48 h, cell was fixed, washed and stained with Sulforhodamine-B stain. Acetic acid was then used to wash excess stain and attached stain was recovered with Tris ethylenediaminetetraacetic acid (EDTA) buffer. Color intensity was measured in an enzyme-linked immunosorbent assay (ELISA). Percentage of the surviving and inhibition were tabulated.

#### 3.4.2. Measurement of Potential EGFR/HER2 Inhibitory Activity (IC_50_)

Measurement of potential EGFR/ HER2 inhibitory activities was performed using Kinase-Glo Plus luminescence kinase assay kit (Promega Corporation, Madison, WI, USA) [25]. Mix kinases, ATP, substrates, and compounds in the reaction buffer of 25 mM HEPES (pH 7.4), 10 mM MgCl_2_, 0.01% Triton X-100, 100 µg/mL BSA, 2.5 mM DTT in a 384-well plate. Total reaction volume was 10 µL. The assay plate was incubated at 30 °C for 1 h, and the reaction was stopped by the addition of equal volume of kinase glo plus reagent. The fluorescence was measured. The signal was correlated with the remaining amount of ATP in the reaction and was inversely correlated with the kinase activity.

### 3.5. Molecular Modeling Study

#### 3.5.1. Protein Preparation for Docking Study

The X-ray crystal structure of the catalytic domain of the EGFR enzyme complex with erlotinib (3) (PDB 1M17 resolution 2.6 A), the human HER2 kinase domain with ligand 03Q (PDB 3PP0 resolution 2.25 A), and EGFR kinase T790M in complex with BIBW2992 (afatinib (5)) (PDB 4G5P resolution 3.17A) were obtained from Protein Data Bank and further prepared by the protein preparation wizards available in the Glide program of Schrodinger 10.1. The preparation portion adds hydrogen after ensuring chemical accuracy and neutralizes side chains that are neither close to the binding cavity nor involved in the formation of salt bridges. For this reason, the OPLS-2005 force field was used, and then the active site of the protein was established. Water molecules were extracted in the next step, and H atoms were added to the crystal structure, most likely hydroxyl and thiol hydrogen atom positions, protonation states, and tautomers of the His residue and chi “flip” assignment were selected by the protein assignment script provided by Schrodinger for Asn, Gln, and His residues. Using the OPLS2005 force field, minimization was carried out to alleviate steric clashes until the average root mean square deviation (RMSD) of the non-hydrogen atoms reached a maximum value of 0.3 A [16,26,27].

#### 3.5.2. Ligand Preparation

All compounds were constructed using the Maestro 10.1 fragment library and prepared using LigPrep 2.1, which can generate several structures with different ionization states, tautomers, stereochemistry, and ring conformations from each input structure. For optimization, the OPLS-2005 force field was used, which generates the ligand’s low-energy conformer. Partial atomic charges were allocated, and potential ionization states were issued at a pH of 7.0. For each ligand, energy minimization was carried out until it met the RMSD cutoff of 0.010 A. The resulting structures were then adopted to carry out modeling studies [27,28].

#### 3.5.3. Molecular Docking

The designed compounds were docked to the EGFR (PDB 1M17), HER2 (PDB 3PP0), and EGFR kinase T790M in complex with BIBW2992 (afatinib (**5**)) (PDB 4G5P resolution 3.17A) binding sites using Glide, grid-based ligand docking with energetics software from Schrodinger 10.1, to test the docking parameters. We scaled the Van der Waals radii of receptor atoms by 0.8 with a partial atomic charge of 0.15 to soften the potential for nonpolar parts of the receptor. At the center of the active site, a grid box with coordinates X = 10, Y = 10, and Z = 10 was created. The ligands were docked with the active site using “extra-precision” glide docking (Glide XP), which flexibly docks ligands. Glide internally creates conformations and passes them through a series of filters. The XP docking technique has been clarified elsewhere. Using a Glide score feature, the final best docked structure was selected. In most comparable docking conformations, the lowest energy docking complex was found. Finally, for further analysis, the lowest energy docked complex was chosen [16].

#### 3.5.4. In Silico ADME Prediction

Drug-like properties of the synthesized compounds were evaluated in accordance with Lipinski’s rule of five ADME. This was used to evaluate whether these compounds have properties that would allow them to be orally active drugs for humans. The drug-like action of our compounds was predicted using module QikProp (v4.2; Schrodinger 2015-1) [16,26].

## 4. Conclusions

New chemical compounds belonging to tetrahydrobenzothieno[2,3-d][1,2,3]triazine, dihydrocyclopentathieno[2,3-d][1,2,3]triazine, and 3-cyanotetrahydrobenzothiophene bearing various heterocyclic systems at positions 4 and 2 were designed, synthesized, structurally elucidated, and biologically evaluated as anti-lung cancer agents. The synthesized compounds exhibited potent in situ cytotoxic activity toward human lung carcinoma cell line H1299 compared to gefitinib (**2**), with IC_50_ values ranging from 12.5 to 54.8 nM. The designed compounds showed antiproliferative activity through competitive inhibition of EGFR and/or HER2 receptors. The designed compound (**21a**) has shown comparable potency to imatinib (**1**) but more potent than neratinib (**6**) in in vitro kinase inhibition against EGFR and HER2. Results of molecular modeling further supported the potent inhibitory activity of the proposed compounds, which helps in understanding the various interactions between ligands and receptor sites. The tetrahydrobenzothieno[2,3-d][1,2,3]triazine scaffold and 3-cyanotetrahydrobenzothiophene occupied the ATP-binding site, showing the same drug–receptor interactions as two approved drugs: lapatinib (**4**) and neratinib (**6**). Additional binding sites, especially in hydrophobic pockets, were provided by our structural modification. The introduction of new functional groups with positions different to those commonly found in approved drugs could allow the designed compounds to interact with alternative binding sites, which could solve the problem of acquired drug resistance. Our new scaffolds might also serve as promising lead compounds for the discovery of new drugs to overcome acquired resistance in NSCLC patients.

## Figures and Tables

**Table 1:** In vitro cytotoxic activity of compounds (**13**–**25a,b**) against non-small cell lung cancer (NSCLC) cell line H1299.

Compound No.	IC_50_ against H1299
**13a**	28.79 nM
**14a**	25.68 nM
**15a**	32.81 nM
**16a**	34.91 nM
**17a**	54.80 nM
**13b**	25 nM
**15b**	34.00 nM
**17b**	38.13 nM
**21a**	12.50 nM
**21b**	13.68 nM
**23**	18.41 nM
**25a**	18.53 nM
**25b**	18.47 nM
Gefitinib (**2**)	40 µM

**Table 2:** In vitro inhibitory activity of compound **21a** against EGFR and HER2 enzymes.

Compound No.	IC_50_ against EGFR (nM)	IC_50_ against HER2 (nM)
(**21a**)	0.47	0.14
Gefitinib (**2**)	1.9	--
Imatinib (**1**)	0.11	0.06
Neratinib (**6**)	92 [21]	59 [21]

**Table 3:** In Silico ADME prediction parameters of designed and reference molecules.

ADME Prediction Parameters	Compound (15a)	Compound (21a)	Lapatinib (4)
mol MW ^a^	371.4	352.1	581.06
Donor-HB ^b^	1	2.25	1
Accept-HB ^c^	5	5.7	8.25
QPlogPo/w ^d^	4.3	2.4	6.3
PSA ^e^	62.2	116.8	95.35
QPlogS ^f^	–5.6	–6.2	–8.2
QPPCaco ^g^	1143.7	96.8	187.5
QPlogBB ^h^	–0.6	–1.9	0.8
QPPMDCK ^i^	1080	59.5	376.5
QPlogKhsa ^j^	0.55	0.26	1.19
#rotor ^k^	5	5	10
% Human Oral Absorption ^l^	100	76.9	78.8

Acceptable ranges: ^a^: mol MW (molecular weight) < 500 amu; ^b^: Donor-HB (hydrogen bond donor) < 5; ^c^: Accept-HB (hydrogen bond acceptor) < 10; ^d^: QPlogPo/w (octanol/water partition coefficient) < 5; ^e^: PSA (Van der Waals surface area of polar nitrogen and oxygen atoms) = 7–200; ^f^: QPlogS (Conformation-independent predicted aqueous solubility) < 0.5; ^g^: QPPCaco (Caco-2 cell permeability) ≤ 5; ^h^: QPlogBB (Predicted brain/blood partition coefficient) <25 poor, h > 500 great; ^i^: QPPMDCK (Predicted apparent MDCK cell permeability in nm/s. MDCK cells are considered to be a good mimic for the blood–brain barrier) < 25 poor, i > 500 great; ^j^: QPlogKhsa (Prediction of binding to human serum albumin) = −1.5 to 1.5; ^k^: rotatable bond = 0–15, ^l^: % Human Oral Absorption (Predicted qualitative human oral absorption) > 80% is high, l < 25% is poor [16].
